# Shaping modern human skull through epigenetic, transcriptional and post-transcriptional regulation of the *RUNX2* master bone gene

**DOI:** 10.1038/s41598-021-00511-3

**Published:** 2021-10-29

**Authors:** Lorena Di Pietro, Marta Barba, Daniela Palacios, Federica Tiberio, Chiara Prampolini, Mirko Baranzini, Ornella Parolini, Alessandro Arcovito, Wanda Lattanzi

**Affiliations:** 1 Dipartimento di Scienze della Vita e Sanità Pubblica, Università Cattolica del Sacro Cuore, Rome, Italy; 2 Fondazione Policlinico Universitario A. Gemelli IRCCS, Rome, Italy; 3 Dipartimento Testa-Collo e Organi di Senso, Università Cattolica del Sacro Cuore, Rome, Italy; 4 Dipartimento di Scienze Biotecnologiche di Base, Cliniche Intensivologiche e Perioperatorie, Università Cattolica del Sacro Cuore, Rome, Italy

**Keywords:** Biochemistry, Biological techniques, Biotechnology, Cell biology, Computational biology and bioinformatics, Evolution, Genetics, Molecular biology, Stem cells

## Abstract

*RUNX2* encodes the master bone transcription factor driving skeletal development in vertebrates, and playing a specific role in craniofacial and skull morphogenesis. The anatomically modern human (AMH) features sequence changes in the *RUNX2* locus compared with archaic hominins’ species. We aimed to understand how these changes may have contributed to human skull globularization occurred in recent evolution. We compared in silico AMH and archaic hominins’ genomes, and used mesenchymal stromal cells isolated from skull sutures of craniosynostosis patients for in vitro functional assays. We detected 459 and 470 nucleotide changes in noncoding regions of the AMH *RUNX2* locus, compared with the Neandertal and Denisovan genomes, respectively. Three nucleotide changes in the proximal promoter were predicted to alter the binding of the zinc finger protein Znf263 and long-distance interactions with other cis-regulatory regions. By surface plasmon resonance, we selected nucleotide substitutions in the 3’UTRs able to affect miRNA binding affinity. Specifically, miR-3150a-3p and miR-6785-5p expression inversely correlated with *RUNX2* expression during in vitro osteogenic differentiation. The expression of two long non-coding RNAs, *AL096865.1* and *RUNX2-AS1*, within the same locus, was modulated during in vitro osteogenic differentiation and correlated with the expression of specific *RUNX2* isoforms. Our data suggest that *RUNX2* may have undergone adaptive phenotypic evolution caused by epigenetic and post-transcriptional regulatory mechanisms, which may explain the delayed suture fusion leading to the present-day globular skull shape.

## Introduction

Evidence of morphological changes between human species, derived from the anatomical study of unearthed bone specimens, enabled confirming selected autapomorphic traits described in extinct hominins^1^. On this regard, the shape of the cranium is among the most relevant features that differ between anatomically modern humans (AMH) and ancient hominins (i.e. Neandertal and Denisovan species). AMH has a more “globular” less elongated skull that, besides other features, displays a more prominent frontal bone, suggesting a different ossification pattern of the neurocranium and a delayed metopic suture fusion, compared with archaic species^2,3^. This globularization of the human skull resulted from an evolutionary path led by and coherent with the structural and functional evolution of the brain, at a pace that has been marked by subtle genetic changes intervened in the human genome.

Upon the sequencing of the entire archaic hominins’ genomes^4–6^, numerous efforts have been devoted to shed light on the evolutionary genetic relationships among species by identifying the genomic changes that may have had functional consequences in the morphological patterning of AMH.

On this regard, the locus containing the *RUNX Family Transcription Factor 2* gene (*RUNX2*) has been identified by Green and collaborators, as part of the genomic regions that underwent a positive selection in AMH after the evolutionary split from Neandertal^4^. The *RUNX2* gene encodes the RunT-related master bone transcription factor, which plays a key role in the osteogenic differentiation process, being essential during the development of the vertebrate skeleton^7^. RunT-related protein (RUNX) coding genes are considered “evolutionary tuning knobs”, whose sequence variations led to key changes in protein functions^8^. Three RUNX gene paralogs are found in vertebrates (*RUNX1*, *RUNX2* and *RUNX3*), with structural homology and conserved protein domains, despite their tissue-specific expression patterns indicating different functions^9,10^. Specifically, *RUNX2* underwent a significant evolutionary pressure that modelled its structure and expression across different vertebrate species. This includes the presence of a unique additional poly-glutamine, poly-alanine tandem repeat domain (QA), which plays a role in protein transactivation^8^ and is thought to be involved in the regulation of membranous ossification and craniofacial growth^11^. *RUNX2* haploinsufficiency causes cleidocranial dysplasia (CCD), a developmental disorder characterized by an altered growth of membranous bones, including hypoplasia or aplasia of the clavicles, delayed closure of cranial sutures and dental anomalies^12–15^. On the other hand, an excessive dosage of *RUNX2*, due to an increase of gene copies, has been linked to craniosynostosis (CS)^16–19^. CS is a congenital malformation due to the premature ossification and fusion of one or more membranous sutures, which leads to a progressive alteration of skull growth patterns and rates in perinatal stages. The ultimate consequence of the abnormal suture ossification in CS, is a skull deformity due to the constraint of skull growth (craniostenosis): skull expansion is restricted along a direction that runs perpendicular to the fused suture(s) while a compensatory growth enhancement occurs along the parallel direction^20^. In around 65% cases CS occurs as an isolated congenital anomaly (nonsyndromic craniosynostosis, NCS), with unclear etiopathogenesis. Instead, syndromic CS has a clearer genetic pathophysiology, entailing wide clinical and genetic heterogeneities, including mutations in over 60 different genes^20,21^. Until very recently, the *RUNX2* locus has been primarily involved in complex syndromic CS, with a clear gene dosage effect, as the phenotype complexity increases with locus duplication, triplication, quadruplication^16–19^. Very recently, our group found nonsynonymous gain-of-function variants of *RUNX2* in NCS affecting midline (i.e. sagittal and metopic) sutures^22^. It is also worth noting that most of the known genes associated with either syndromic or nonsyndromic CS are involved in largely interacting pathways that merge on RUNX2 as the main downstream effector of the osteogenic cascade^20^.

*RUNX2* gene might have been involved in the molecular regulation path underneath the recent evolution of anatomically modern humans (AMH) from archaic hominin species^4^. Being specifically associated with quantitative traits and congenital defects affecting skull morphogenesis (*i.e.* CCD and CS), this gene might indeed represent an interesting evo-devo correlate reference in this context.

**Figure 1:**
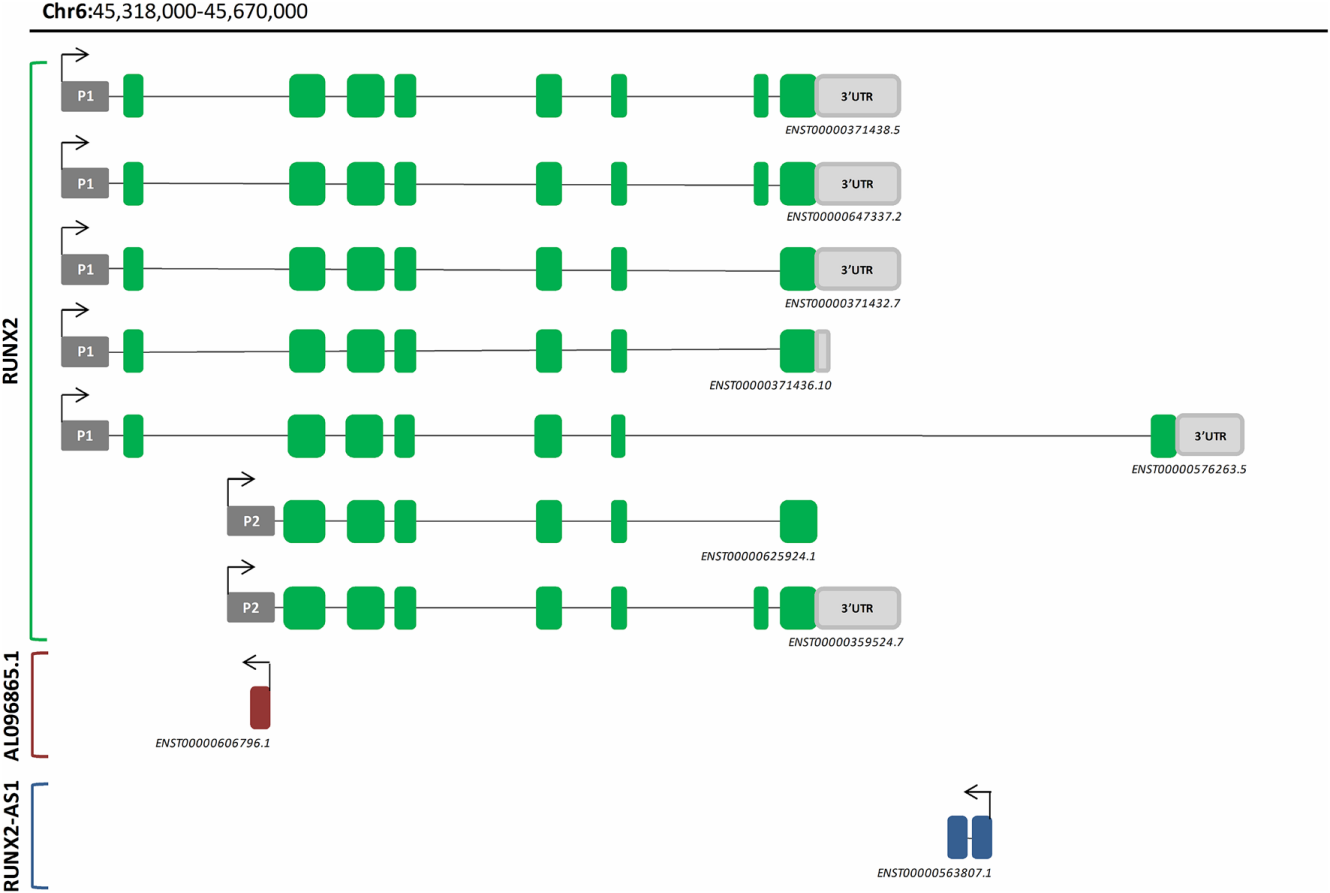
RUNX2 gene locus. The diagram outlines the structure and exon distribution of the different *RUNX2* coding isoforms and of the two lncRNA antisense to *RUNX2* (*AL096865.1* and *RUNX2-AS1*), all derived from the transcription of the *RUNX2* gene locus on chromosome 6 (source https://www.ensembl.org/). The upper part of the figure lists the seven coding *RUNX2* transcripts, with the exons coloured in green and the untranslated regions (UTR) at the 3’ end coloured in light grey. The arrows on the promoters (P1: Promoter 1, P2: Promoter 2) indicate the direction of the transcription.

Based on the genetic data available to date, the human *RUNX2* gene encodes 7 different protein isoforms, plus 3 isoforms subjected to nonsense-mediated decay, and 2 classified as processed transcripts, resulting from the activation of two alternative promoters (the distal promoter 1, P1 and the proximal promoter 2, P2), and from alternative splicing^23^, as outlined in Fig. 1. In mice, P1 is activated during osteogenesis and is mandatorily required for bone formation, while P2 is expressed in both osseous and non-osseous mesenchyme, and is thought not to be exclusively linked to osteogenesis^24,25^. Our previous findings highlighted how the expression patterns of *RUNX2* P1- and P2-isoforms are related with the timing of calvarial sutures closure affecting the regulation of the skull shape in humans^22^.

*RUNX2* transcripts differ also in the untranslated regions (UTRs), as they may feature two alternative 3’-UTRs (the proximal 3’UTR and the distal 3’UTR; Fig. 1). Only a single isoform contains the distal 3’UTR, and is also characterized by the presence of a distal translated exon, which is missing in all the other coding isoforms (Fig. 1).

None of the nucleotide changes that were positively selected in AMH falls within the coding region of *RUNX2* open reading frame, though many were found within noncoding regions throughout the gene locus^4^. It has been hypothesized that these fixed sequence changes could have affected regulatory sequences, hence they could have modified *RUNX2* expression and functional status, changing the timing of suture fusion in skull morphogenesis and leading to a more globular shape in AMH^26^.

Based on this background, the aim of our study was to comparatively characterize the genomic structure of *RUNX2* locus in AMH and extinct hominins (Neandertal and Denisovan), and to derive functional correlations between *RUNX2* expression regulation and skull morphogenesis.

## Results

### Study of RUNX2 locus in modern and ancient humans

**Figure 2:**
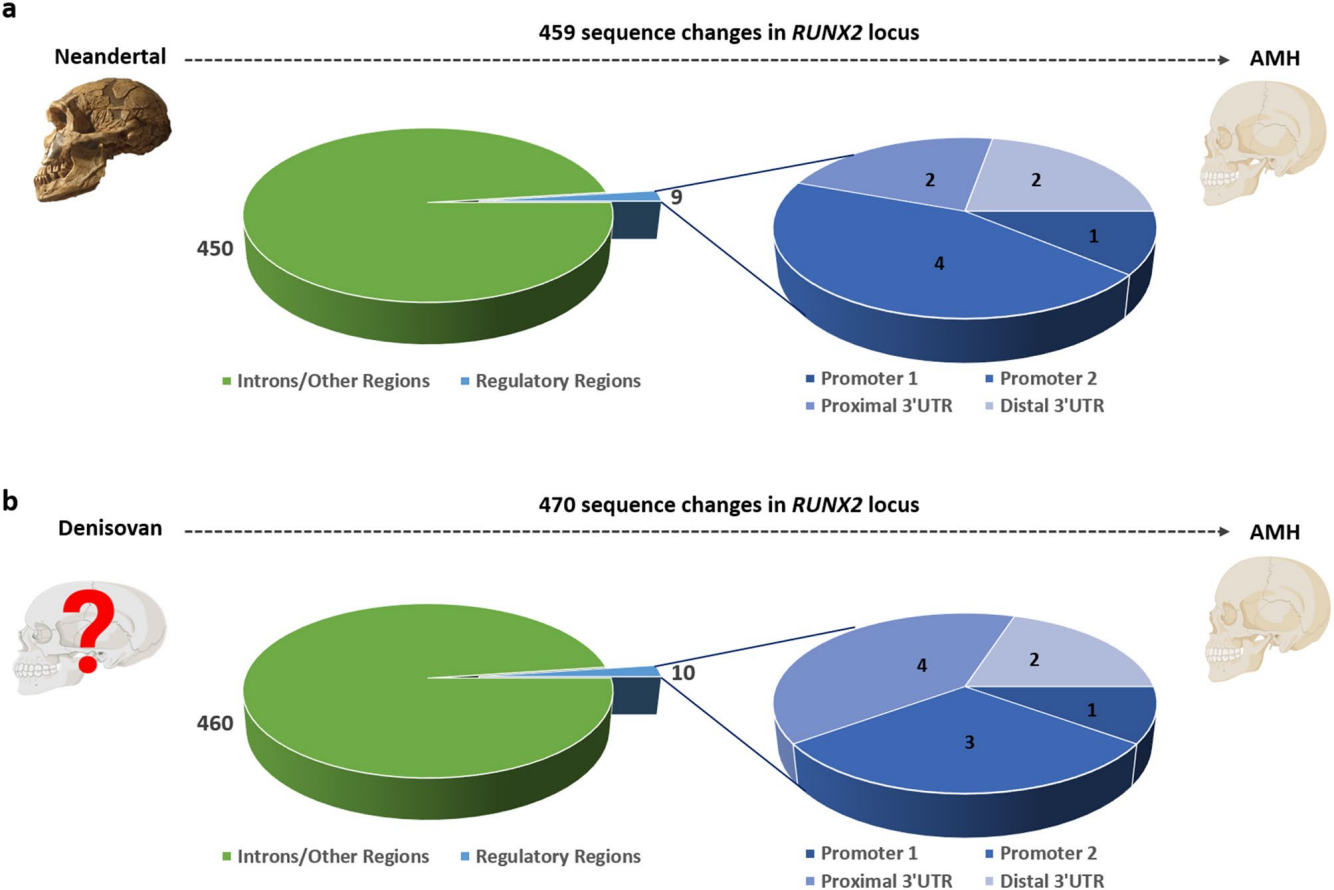
*Divergence of RUNX2 locus in AHM compared with ancient hominins. *The pie charts display the extent of genomic divergence in terms of number of sequence changes and corresponding locations found in AMH compared with the Neandertal **(a)** and the Denisovan **(b)** species identified using the Integrative Genomics Viewer (IGV) tool. Most of the sequence changes are found in intronic regions, whereas few substitutions occur in the regulatory regions outside the open reading frame: Promoter 1, Promoter 2, proximal 3’UTR and distal 3’UTR. The Neandertal skull in the upper left panel of this figure is exhibited at the Natural History Museum of London (personal picture, credits to W. Lattanzi); the AMH’s and Denisovan’s skulls were created with BioRender.com (note that a reliable reconstruction of the Denisovan skull is not available).

The sequences of the *RUNX2* locus (Chr6:45,318,000–45,670,000 hg38, for a total of 352.000 bases) have been aligned in AMH and ancient species’ (Neandertal and Denisovan) genomes to map the changes occurred during recent evolution. We have identified 459 and 470 changes (including nucleotide substitutions, indels, and annotated human SNPs) acquired in AMH compared with Neandertal and Denisovan, respectively (Supplementary File 1 and Fig. 2). Our analysis confirmed that no change occurred within *RUNX2* exons. Selected changes map within the P1 and P2 promoter sequences, and in both proximal and distal 3’UTRs (Fig. 2). In addition, to confirm which of these nucleotide changes have occurred during the recent human evolution, we also analysed the *RUNX2* sequences of non-human Primates [chimpanzee (*Pan troglodytes*), gorilla (*Gorilla gorilla*), Sumatran orangutan (*Pongo abelii*), gibbon (*Nomascus leucogenys*) and rhesus macaque (*Macaca mulatta*)] focusing our attention on changes mapping in promoters and 3’UTRs (Supplementary File 1).

The P1 sequence in AMH differs for as little as one nucleotide from Neandertal and Denisovan genes (Supplementary File 1 and Fig. 2). In the P2 sequence, we found three changes in AMH compared with both ancient hominins, plus an additional change differing exclusively between AMH and Neandertal (Supplementary File 1 and Fig. 2). Interestingly, three substitutions mapping in the P2 promoter were not present in any of the tested Primates species, which retained the same nucleotides observed in the ancient hominins’ species. These changes seem indeed to be specific to AMH (Supplementary File 1).

The proximal 3’UTR sequence of AMH differs in 4 and 2 nucleotides, compared to Neandertal and Denisovan species, respectively (Supplementary File 1 and Fig. 2). Our analysis revealed that one change in the proximal 3’UTR identified in Denisovan *RUNX2* sequence is also conserved in all non-human Primates analysed (Supplementary File 1). The sequence of the distal 3’UTR region of AMH, instead, differs from both Neandertal and Denisovan due to two nucleotide substitutions (Supplementary File 1 and Fig. 2). All the tested Primates’ genome sequences differ from both modern and ancient humans in one of these changes (Supplementary File 1).

*RUNX2* locus also includes two additional genes, recently classified as lncRNAs antisense to *RUNX2*, namely, *AL096865.1* and *RUNX2 antisense RNA 1* (*RUNX2-AS1*) (Fig. 1). *AL096865.1* partially overlaps with the *RUNX2* P2 region and contains 3 sequence changes that occurred in the AMH genome compared with both Neandertal and Denisovan (i.e. the same changes already described for P2 sequence; Supplementary File 1). *RUNX2-AS1* encodes a transcript (*ENST00000563807.1*) with two exons, and maps on the reverse strand of the last intron of the *RUNX2 ENST00000576263*.5 coding isoform (see Fig. 1). Our results showed that 6 changes map within the *RUNX2-AS1* gene, 2 of which fall in the first exon (Supplementary File 1). Three of the substitutions mapping in the *RUNX2-AS1* locus were not found in the tested Primates’ genomes, which shared the same nucleotides observed in the ancient hominins (Supplementary File 1).

### Nucleotide changes in *RUNX2* proximal promoter localize at ZNF263 binding sites

To assess the putative functional consequence of the sequence changes observed in the *RUNX2* gene regulatory regions, we have performed in silico predictive analysis.

We first focused on the analysis of nucleotide substitutions within the two promoter regions, P1 and P2. As discussed above, P1 sequence in AMH differs from that of Neandertal and Denisovan for just 1 nucleotide. Downstream analysis using different bioinformatic tools (Meme suite https://meme-suite.org/meme/, Consite http://consite.genereg.net/ and rVista 2.0 https://rvista.dcode.org/)^27–29^ did not reveal any association of this genomic substitution with putative regulatory regions.

**Figure 3:**
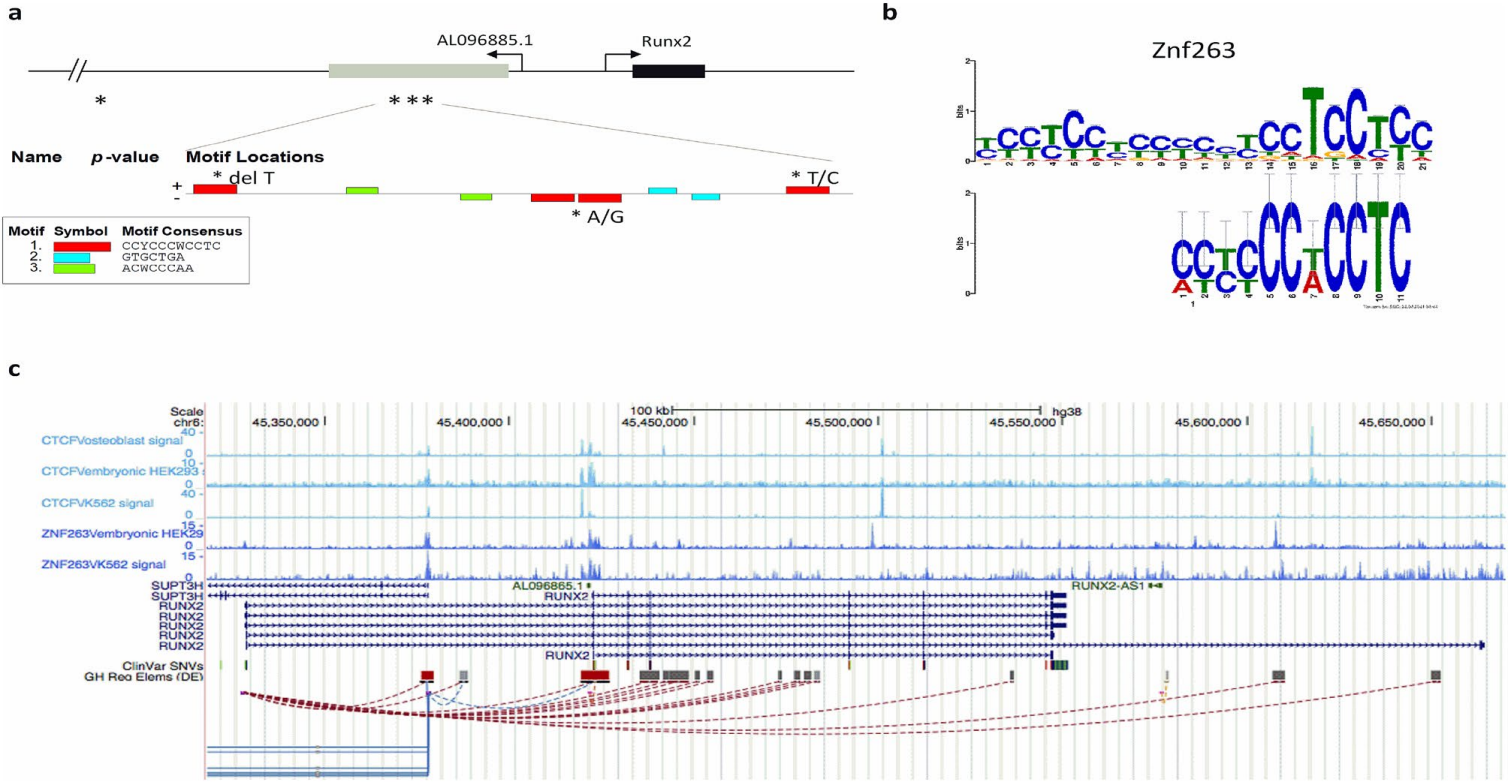
*Genomic variants in RUNX2 P2 promoter overlap with ZNF263 binding sites at CTCF-mediated loop anchor regions. (a)* Motif analysis using MEME suite of the P2 proximal promoter region, containing a cluster of three nucleotide variants in AMHs compared with Denisovan/Neandertal species (chr6:45,421,398–45,421,567), identified three CCYCCCWCCTC motifs overlapping with the three genomic variants. **(b)** In silico analysis using Tomtom identified ZNF263 as a transcription factor binding the CCYCCCWCCTC motif. **(c)** Scheme of the *RUNX2* locus (Chr6:45,318,000–45,670,000 GRCh38/hg38) showing the different *RUNX2* isoforms and regulatory regions and available Encode data. These include: ChIP-seq tracks (fold change over control) for CTCF binding (light blue) in osteoblasts, HEK293 and K562 cell lines, ChIP-seq tracks (fold change over control) for Znf263 binding (dark blue) in HEK293 and K562, short nucleotide clinical variants ClinVar SNVs and GeneHancer regulatory elements (GH Reg Elem (DE)), including 3D chromatin loop formation.

For P2, we found four nucleotide changes, three differing in AMH compared with both ancient hominins, and 1 differing exclusively between AMH and Neandertal. Three of these substitutions also overlap with the antisense lncRNA *AL096865.1* (Supplementary File 1)*.* Motif analysis using MEME suite^27^ indicated that 3 substitutions map within 3 conserved CCYCCCWCCTC sequence motifs (Fig. 3a, red boxes). Interestingly, analysis of transcription factor binding using Tomtom^30^ identified this motif as a putative binding site for the C2H2-type zinc finger protein ZNF263 (Fig. 3b). We therefore applied position weigh matrix (PWM) analysis to investigate if the identified substitutions could affect binding of this transcription factor to *RUNX2* P2 promoter. Analysis of position-specific scoring matrices using available software (FIMO, Find Individual Motif Occurrences, https://meme-suite.org)^31^ indicated that: (i) two of these substitutions (A/G in position 45421497 and T/C in position 45421552) decreased ZNF263 matrix score in AMHs from 17.102 to 15.734, and from to 6.637 to 4.959, respectively, while (ii) the inclusion of a T in Denisovan and AMHs (position 45421406) increases the matrix score from 4.204 to 4.653 (Supplementary File 2). ZNF263 was recently shown to participate in the CCCTC binding factor (CTCF)-mediated chromatin looping^32^ and its motif is also enriched in lncRNAs located at chromatin boundaries^33^. As chromatin looping was previously associated with the regulation of *RUNX2*^34^, we hypothesize that the tested nucleotide changes could alter chromatin looping and therefore influence the levels of gene expression. To support this hypothesis, we first investigated if P2 promoter was indeed bound by CTCF and ZNF263. To this end, we explored available ENCODE (https://www.encodeproject.org/) genome-wide datasets from different human cell lines (osteoblasts, HEK293 and K562 cells), using the UCSC Genome browser resources (https://genome.ucsc.edu). Data shown in Fig. 3c show that CTCF binding is enriched at the P2 promoter in all cell lines analysed, whereas ZNF263 binding was found in the two cell lines for which there were ENCODE data available (HEK293 and K562). Finally, analysis of the GeneHancer database revealed different loop interactions involving the *RUNX2* P2 promoter and other regulatory elements in the region, such as SUPT3H (SPT3 homolog, SAGA and STAGA complex component) promoter (Fig. 3c). Altogether, these analyses suggested that the genomic changes at the ZNF263 binding sites comprised within the *RUNX2* P2 promoter/*AL096865.1* lncRNA sequence could influence DNA looping and therefore differentially regulate *RUNX2* expression in AMH as compared with ancient hominins.

### Post-transcriptional regulatory effects of nucleotide substitutions in the two 3’UTRs of *RUNX2*

We then focused further analyses on the study of the nucleotide substitutions within the regulatory elements at the 3’ ends of *RUNX2*, to evaluate possible implications in post-transcriptional regulation.

Using the UCSC Genome browser resources, we observed that the proximal 3’UTR belongs to a hotspot region of variants annotated in the ClinVar database (https://www.ncbi.nlm.nih.gov/clinvar/; see Fig. 3c). As mentioned above, the proximal 3’UTR sequence of AMH differs in six nucleotides respect to ancient hominis (in particular, four respect to Neanderthal and two nucleotides compared to Denisovan). Of these, four nucleotides specific for ancient hominis represent common variants annotated in the Homo sapiens genome (rs144321470, rs188598788, rs537488922, rs182124295), as ‘benign mutations’ in CCD cohorts. Also, the variants in the distal 3’UTR of Neandertal and Denisovan correspond to common variants observed in Homo sapiens (rs74697776, rs6458457) that are not annotated in ClinVar.

The 3'UTR of a mRNA can be bound by miRNAs that modify the stability and half-life of the target transcripts and/or protein translation. We thus tested the hypothesis that the nucleotide changes found in the 3’UTRs of *RUNX2* could influence miRNA binding. We first used different online databases and tools (miRDB http://mirdb.org/, TargetScanHuman http://www.targetscan.org/vert_72/, miRbase http://www.mirbase.org/ and PolymiRTS Database 3.0 http://compbio.uthsc.edu/miRSNP/), to select the list of miRNAs that, based on sequence complementarity, most likely bind the *RUNX2* 3’UTRs. Then, we annotated all the miRNAs whose seed sequence fall into one of the sequence regions that diverge between AMH and ancient hominins.

**Figure 4:**
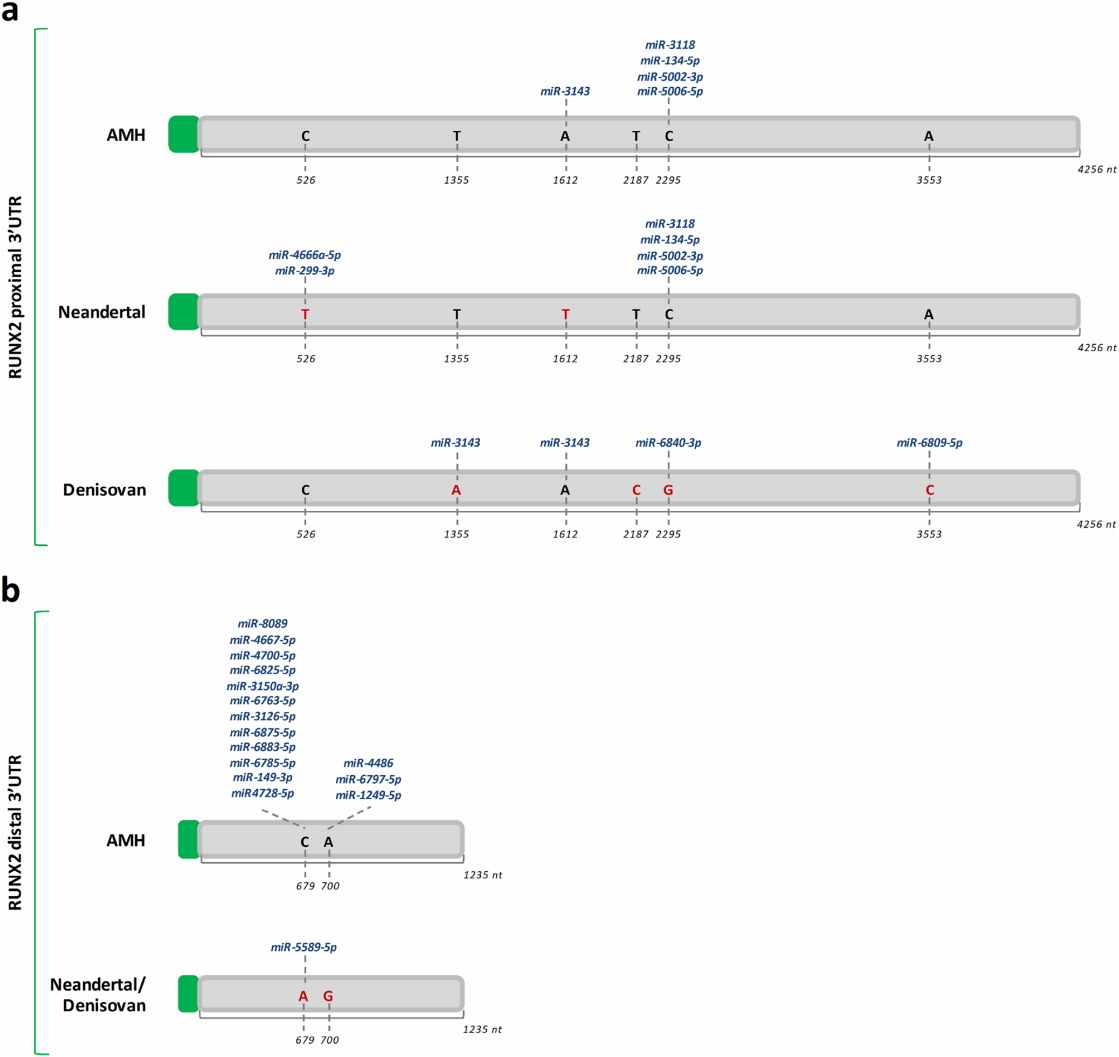
In silico prediction of miRNA binding sites within RUNX2 3’UTRs in AMH, Neandertal and Denisovan. The picture shows the nucleotide changes (highlighted in red) identified in the untranslated regions (UTRs) at the 3’ ends of *RUNX2* transcripts in Neandertal and Denisovan hominins compared with that of anatomically modern humans (AMH). The substitutions are apparently able to modify the pattern of miRNA binding both in the proximal 3’UTR **(a)** and in the distal 3’UTR (**b)**.

Our results showed that the substitutions fixed in the proximal 3’UTR of *RUNX2* affected the binding of different miRNAs, indicating that during recent human evolution, the AMH *RUNX2* genomic region acquired or lost miRNA binding sites, potentially involved in post-transcriptional regulation (Supplementary File 3 and Fig. 4a).

Our data showed also that the distal 3’UTR does not differ between Neandertal and Denisovan species, suggesting a similar miRNA-dependent post-transcriptional regulation pattern. We predicted that the sequence divergence between AMH and ancient hominins observed in this 3’UTR fall within the binding sites of 16 different miRNAs in AMH (Supplementary File 3 and Fig. 4b). In particular, the in silico analysis suggested that the 2 nucleotide changes that were fixed in AMH generate new binding sites for several miRNAs (Fig. 4b). The first substitution in the distal 3’UTR found in the ancient hominins instead should allow the binding of an additional miRNA, namely miR-5589-5p (Fig. 4b).

All the genomic loci of the selected miRNAs were comparatively analysed in the aligned sequences of AMH and ancient hominins, using the IGV tool, to evaluate the evolutionary conservation of their DNA sequences across human species. Our analysis showed that nucleotide changes occurred in the AMH miR-3143, compared with the Denisovan sequence, and in the AMH miR-149-3p, compared both with Neandertal and Denisovan species (Supplementary File 4). Nonetheless, both these fixed changes fall outside the seed sequences of these two miRNAs. All the other miRNAs did not present any nucleotide change.

### RUNX2 isoforms expression in calvarial mesenchymal stromal cells

To delve into functional aspects, we have assessed the differential contribution of the alternative transcript isoforms of the *RUNX2* gene to the osteogenic cascade activation. To this end, we have analysed the expression of the different *RUNX2* transcript variants in mesenchymal stromal cells isolated from calvarial sutures (CMSC) isolated from unfused suture tissues obtained as surgical waste from surgery of nonsyndromic craniosynostosis (NCS) patients. We relied on this cellular model as a benchmark for the study of membranous ossification, previously optimized and standardized in our lab^35^. P1-derived transcripts, P2-derived transcripts, splice variants containing the proximal 3’UTR and the distal 3’UTR, were independently amplified by Real-Time PCR using isoform-specific oligonucleotide primer pairs.

**Figure 5:**
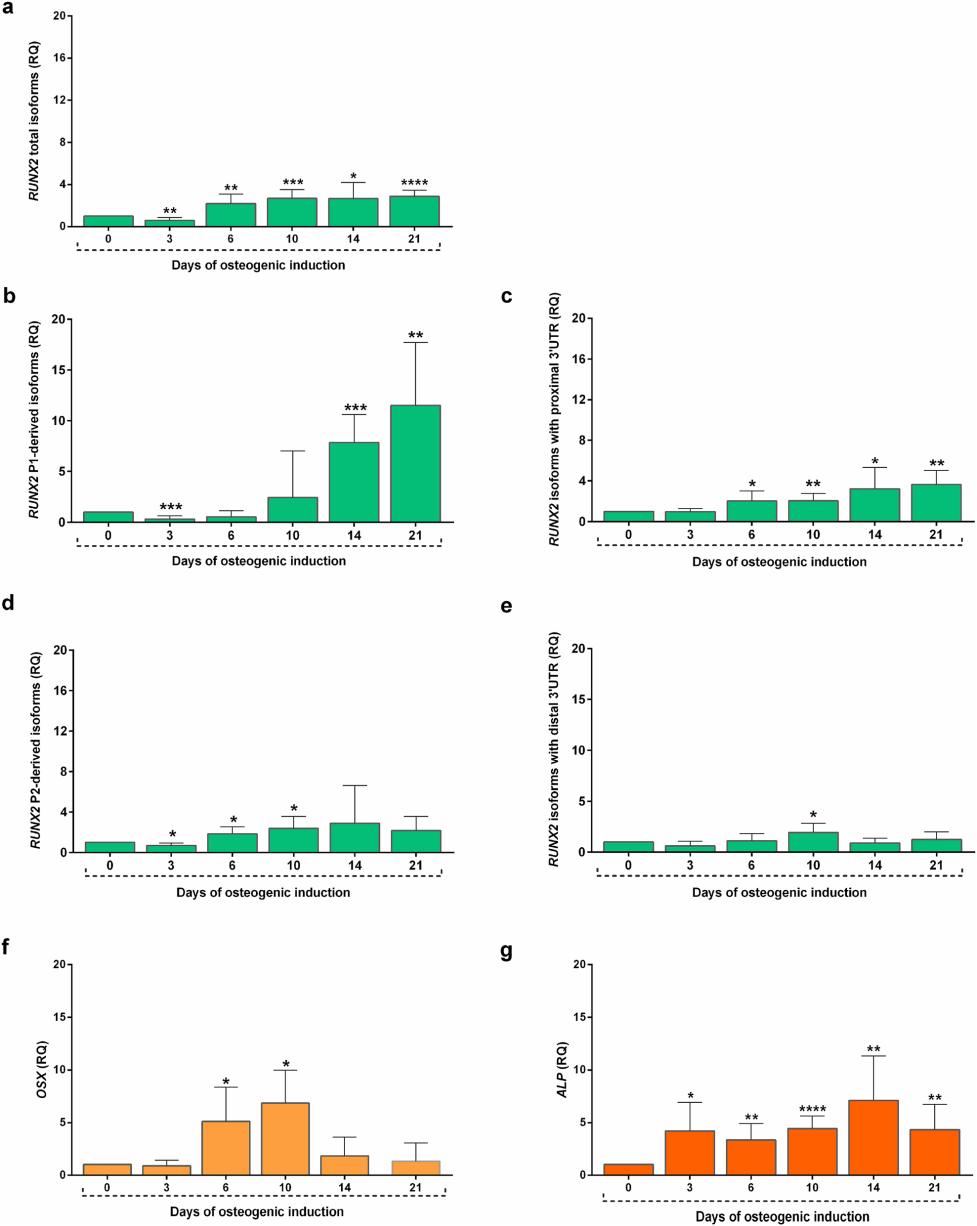
Relative expression of RUNX2 isoforms during osteogenic induction in vitro. The graphs show the expression of total *RUNX2* levels **(a)**, *RUNX2* P1-derived transcripts **(b)**, *RUNX2* splice variants containing the proximal 3’UTR **(c)**, P2-derived transcripts **(d)**, isoforms with the distal 3’UTR **(e)**, *OSX* **(f)** and *ALP* **(g)** transcripts measured by Real Time PCR, in cells undergoing osteogenic induction up to 21 days. The fold change of expression levels is expressed as “relative quantity” (RQ), calculated according to the ΔΔCt method, setting the day 0 (when the standard growth medium has been replaced with the osteogenic medium) value as reference. All data are expressed as mean fold change ± standard deviation across replicates. *p ≤ 0.05; **p ≤ 0.01; ***p ≤ 0.001; ****p ≤ 0.0001.

CMSC were induced toward the osteogenic lineage up to 21 days in order to assess the regulation of each transcript group during the in vitro differentiation process at different timepoints (i.e. 3, 6, 10, 14 and 21 days). Our results showed that total *RUNX2* levels undergo an initial down-regulation during the first days of commitment, followed by an increase thereafter (Fig. 5a). The upregulation of the *RUNX2* P1-derived isoforms and of the *RUNX2* isoforms containing the proximal 3’UTR was significant until 21 days of osteogenic induction (Fig. 5b,c). Instead, the expression of *RUNX2* P2-derived isoforms and of the *RUNX2* isoforms with the distal 3’UTR appeared to reach a peak at 10 days of in vitro differentiation and thereafter decrease or lose statistical significance (Fig. 5d,e). The expression of the Sp7 transcription factor gene (osterix, *OSX*) and of the alkaline phosphatase (*ALP*) gene were evaluated to confirm that the cells were differentiating along the osteogenic lineage (Fig. 5f,g).

### RUNX2-associated lncRNA expression in calvarial mesenchymal stromal cells

**Figure 6:**
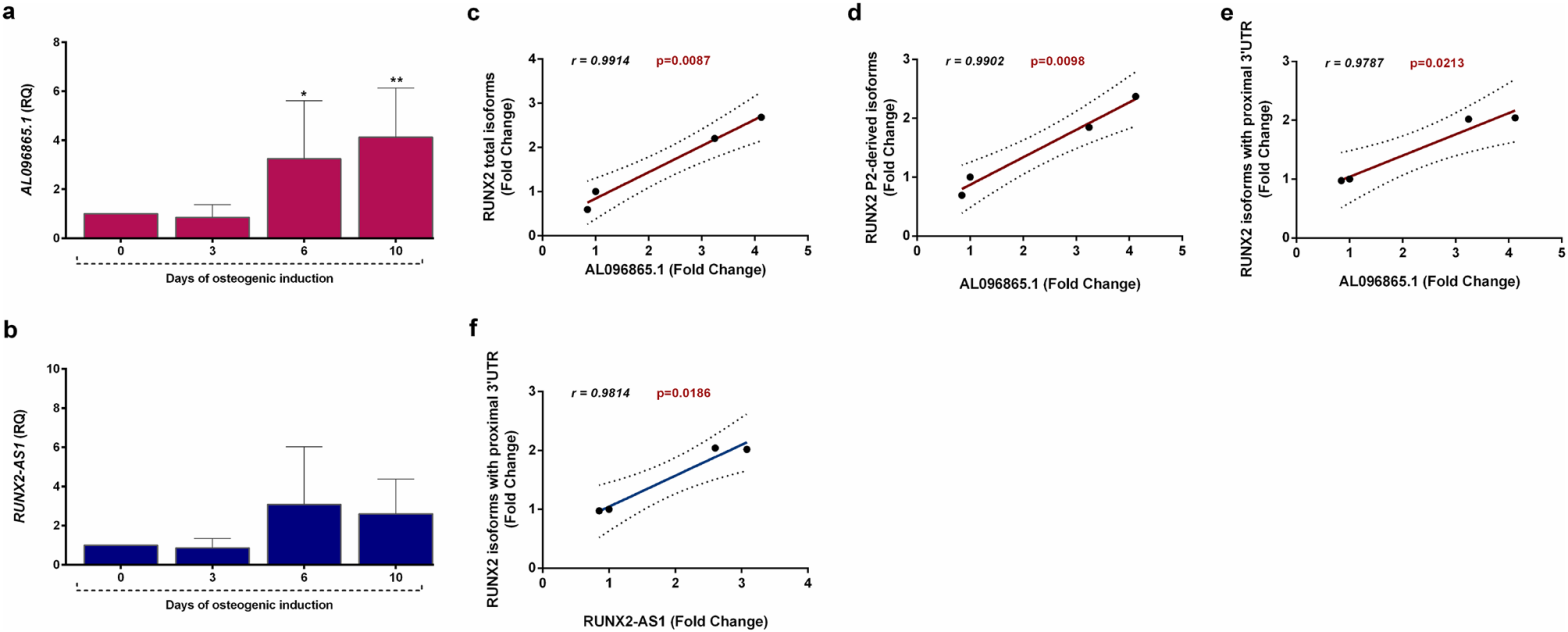
*lncRNAs expression during osteogenic induction *in vitro*.* **(a,b)** The bar graphs show the expression of *AL096865.1* **(a)** and *RUNX2-AS1* **(b)** measured by real time PCR, in cells undergoing osteogenic induction up to 10 days. The fold change of expression levels is expressed as “relative quantity” (RQ), calculated according to the ΔΔCt method, setting the day 0 (when the standard growth medium has been replaced with the osteogenic medium) value as reference. All data are expressed as mean fold change ± standard deviation across replicates. *p ≤ 0.05; **p ≤ 0.01. **(c–f)** The line graphs draw the linear regressions during the first phases of osteogenic induction (up to 10 days) of the relative expression levels of *AL096865.1* with total *RUNX2* levels **(c)**, *RUNX2* P2-derived transcripts **(d)** and *RUNX2* splice variants containing the proximal 3’UTR **(e)**, and of *RUNX2-AS1* with the group of *RUNX2* isoforms containing the proximal 3’UTR **(f)**. Pearson correlation was evaluated and the Pearson correlation coefficient (r value) and p-value (p) were reported.

The expression of the two long noncoding transcripts *AL096865.1* and *RUNX2-AS1* was also assessed in CMSC and correlated to the levels of *RUNX2*, to evaluate the possible involvement of these two lncRNAs in regulating *RUNX2* expression. We focused our analysis during the early stages of the osteogenic commitment (0-to-10 days of osteogenic induction), as a functionally relevant timeframe for gene expression regulation. *AL096865.1* levels were significantly upregulated after 6 and 10 days of differentiation (Fig. 6a); also *RUNX2-AS1* levels showed an increasing trend during osteogenic differentiation (Fig. 6b). Furthermore, our analysis showed a significant positive correlation between the expression of *AL096865.1* and that of *RUNX2* total isoforms (Fig. 6c), of *RUNX2* P2-derived isoforms (Fig. 6d) and of *RUNX2* isoforms containing the proximal 3’UTR (Fig. 6e). A significant positive correlation could be also outlined for *RUNX2-AS1* and the *RUNX2* isoforms containing the proximal 3’UTR (Fig. 6f). These data might suggest a feasible involvement of *AL096865.1* and *RUNX2-AS1* in modulating *RUNX2* expression.

### Modelling the interaction between miRNAs and RUNX2 isoform with the distal 3’UTR

We further focused our analysis on the distal 3’UTR of *RUNX2* (*ENST00000576263.5*)*,* given the structural peculiarity of this gene, which includes an additional coding exon (see Fig. 1), and taking into consideration that the nucleotide substitutions identified in the proximal 3’UTR were annotated as clinical variants (though benign, to date).

**Figure 7:**
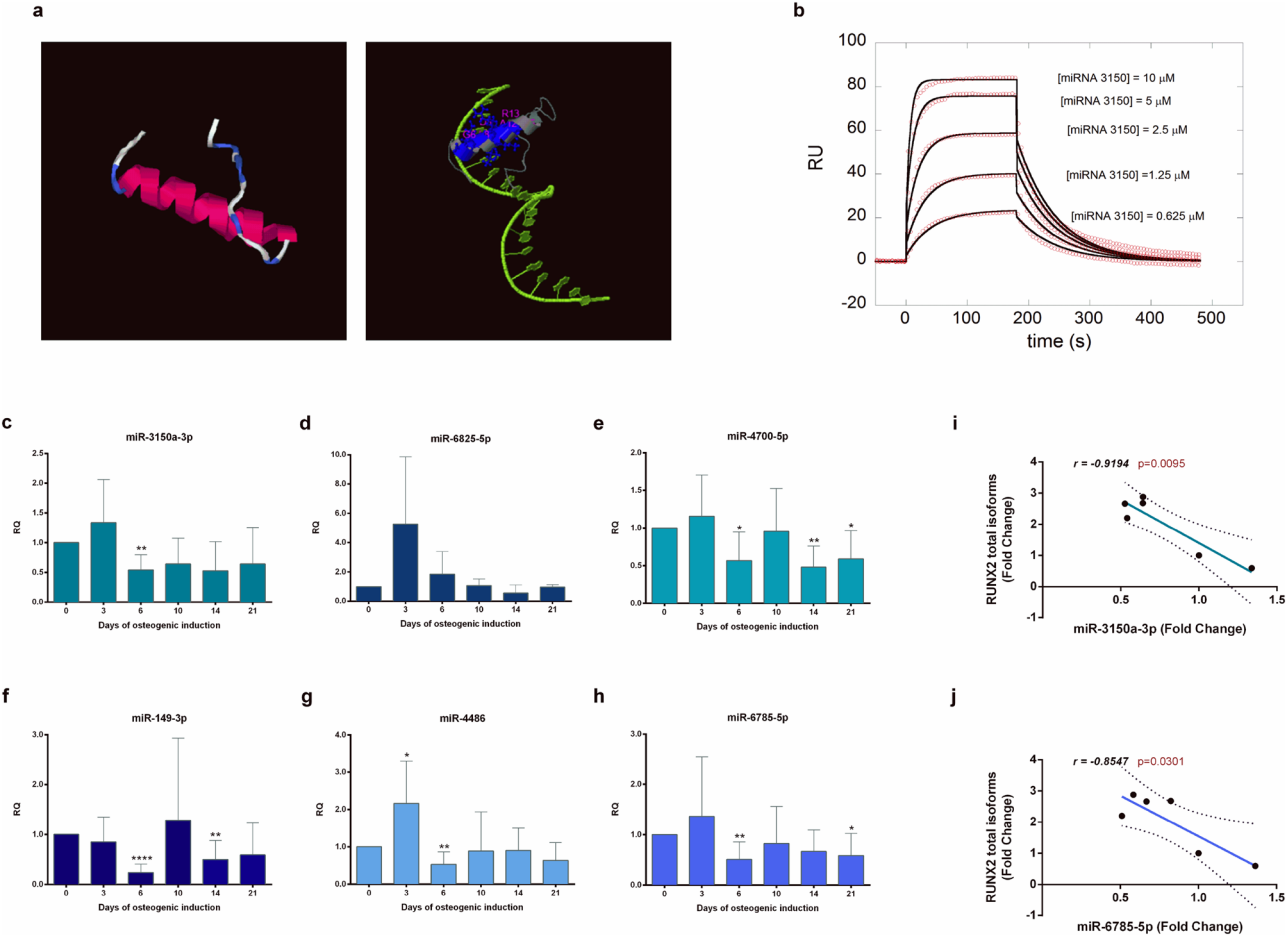
In silico modelling and miRNA profiling. **(a)** The images show the three-dimensional computational modelling of the last coding exon of the RUNX2 isoform containing the distal 3’UTR and its predicted interaction with DNA, by I-TASSER tool (https://zhanglab.ccmb.med.umich.edu/I-TASSER/). **(b)** Surface plasmon resonance data showed the changes in miRNA-target binding affinities. Representative graph showing the interaction of the synthetic sequence that mimics the miR-3150a-3p with the 3’UTR of anatomically modern human (AMH) at different concentration (RU: response unit). Empty circles represent the experimental traces at different analytes’ concentration, while the black lines are the global fit using a 1:1 kinetic model. **(c–h)** The bar graphs show the changes in relative expression of miR-3150a-3p, miR-6825-5p, miR-4700-5p, miR-149-3p, miR-4486 and miR-6785-5p measured by Real Time PCR, in cells undergoing osteogenic induction up to 21 days. The fold change of expression levels is expressed as “relative quantity” (RQ), calculated according to the ΔΔCt method, setting the day 0 (when the standard growth medium has been replaced with the osteogenic medium) value as reference. All data are expressed as mean fold change ± standard deviation across replicates. *p ≤ 0.05; **p ≤ 0.01; ****p ≤ 0.0001. **(i,j)** The line graphs show the linear regressions during the osteogenic induction (up to 21 days) between the relative expression levels of miR-3150a-3p and miR-6785-5p and *RUNX2* total isoforms. Pearson correlation was analysed and the relative Pearson correlation coefficient (r value) and p-value (p) were reported.

In silico protein modelling, based on the I-TASSER tool, revealed that the presence of this terminal exon introduces a leucine-zipper DNA binding motif in the protein (Fig. 7a), likely affecting RUNX2 function as a transcriptional regulator.

We thereafter investigated in vitro if the nucleotide changes in this 3’UTR actually affect miRNA binding. To this end, we used Surface Plasmon Resonance (SPR) to determine the affinity of the molecular interactions between the 15 selected miRNAs and the target sequences on the distal 3’UTR of AMH and Neandertal/Denisovan *RUNX2* genes. The resulting dissociation constant values obtained for each tested miRNA are reported in Table 1. Of the 15 miRNAs tested, eight failed to show any interaction. The remaining seven miRNAs showed differential binding affinities to the species-specific *RUNX2* transcripts: (i) namely, miR-4700-5p, miR-6825-5p, miR-3150a-3p, miR-6763-5p showed interaction exclusively with the AMH *RUNX2* gene; (ii) miR-149-3p showed a higher affinity for the AMH *RUNX2* mRNA; and (iii) miR-6785-5p and miR-4486 showed higher affinity in the interaction with the ancient species’ target sequence (see Table 1). A representative figure corresponding to the interaction of the *RUNX2* 3’UTR of AMH with the synthetic sequence that mimics the miR-3150a-3p is reported in Fig. 7b.

**Table 1:** Measure of the affinity by surface plasmon resonance.

MicroRNA	AMH KD* (µM)	Neanderthal/Denisovan KD^a^ (µM)
miR-8089	No interaction	No interaction
miR-4667-5p	No interaction	No interaction
miR-4700-5p	1.5 ± 0.2	No interaction
miR-6825-5p	5 ± 1	No interaction
miR-3150a-3p	1.23 ± 0.08	No interaction
miR-6763-5p	4.0 ± 0.1	0.92 ± 0.02
miR-3126-5p	Not defined	Not defined
miR-6875-5p	No interaction	No interaction
miR-6883-5p	No interaction	No interaction
miR-6785-5p	7.7 ± 2	2.5 ± 0.3
miR-149-3p	3.3 ± 0.3	13.0 ± 1.5
miR-4728-5p	No interaction	No interaction
miR-4486	2.7 ± 0.5	No interaction
miR-6797-5p	No interaction	No interaction
miR-1249-5p	No interaction	No Interaction

^a^KD: dissociation constants for each selected miRNA with the target sequences on the distal 3’UTR of AMH and Neandertal/Denisovan *RUNX2* genes.

### Analysis of miRNA levels during osteogenic differentiation

To validate these data at the functional level in the biological context, we have analysed in CMSC the levels of the miRNAs previously selected through SPR (miR-3150a-3p, miR-6825-5p, miR-4700-5p, miR-149-3p, miR-4486 and miR-6785-5p). Our results showed that all the miRNAs are expressed in CMSC and that their expression vary during osteogenic differentiation. The expression levels measured during the osteogenic induction were variable across the replicates and it was not always possible to identify a clear trend of activation/silencing during the osteogenesis process (Fig. 7c–h). Due to this technical limitation, we could not detect any significant correlation between the trend of expression of the selected miRNAs and that of the *RUNX2* isoform with the distal 3’UTR during the osteogenic induction (data not shown). However, the expression levels of miR-3150a-3p and of miR-6785-5p inversely correlated with those of *RUNX2* total isoforms: while the expression of *RUNX2* increased during the osteogenesis in vitro, the levels of miR-3150a-3p and miR-6785-5p decreased (Fig. 7i,j). This may suggest that these two miRNAs could have gained a function as post-transcriptional regulators of *RUNX2* in AMHs, tampering excessive production of this transcription factor and delaying ossification.

## Discussion

Changes in gene regulation, owing to stable variants enriched in cis-regulatory regions of key developmental genes, are widely recognized as important drivers of adaptive phenotypic evolution^36,37^.

Our data suggest that *RUNX2* genomic evolution may have led to changes in the expression regulation of this gene, probably affecting the three-dimensional structure of the locus, the binding of selected miRNAs, and the function of two newly discovered lncRNAs antisense to *RUNX2*.

Boeckx and Benitez-Burraco previously hypothesized that a different RUNX2 dosage resulting from the nucleotide changes found in the promoter sequences in AMH compared with ancient hominins, would explain the structural differences in skull morphogenesis^38,39^.

The distal *RUNX2* promoter (P1) is specifically required for endochondral bone formation (occurring from cartilage buds of the skeleton and involved in limb patterning and morphogenesis), as demonstrated in knockout studies^40^. The knockout of the entire gene, including the proximal promoter (P2), leads to impaired skeletal ossification also in membranous bones, like those forming the cranium and the clavicle^40^. Interestingly, *Runx2* isoforms expressed from P2 have been proved to be expressed in cranial sutures^24^. We have previously demonstrated that *RUNX2* expression, and particularly the P1-isoform group, are overexpressed in the osteogenic precursor cells (CMSC) isolated from prematurely fused sutures of nonsyndromic craniosynostosis patients^22^.

In the present study, we have mapped over 900 sequence variations that have occurred in noncoding regions of the *RUNX2* locus of AMH compared with ancient hominin species. Of these, 1 and 4 fixed nucleotide changes map within the P1 promoter and the P2 promoter regions, respectively. Several nucleotide changes found within the promoters and 3’UTR regions of *RUNX2* occurred during the most recent evolution, given that the nucleotides found at those positions in ancient hominins appeared to be conserved across non-human Primates species (*Pan troglodytes*, *Gorilla gorilla*, *Pongo abelii*, *Nomascus leucogenys* and *Macaca mulatta*). This observation may further support a putative role for *RUNX2* in human skull globularization.

Our results showed that, in particular, substitutions in the P2 promoter map within a predicted binding site for the ZNF263 transcription factor, which regulates chromatin structure through DNA looping at CTCF-bound regions^32^. We have indeed found that both ZNF263 and CTCF binding motifs were enriched at *RUNX2* P2 in different cell lines annotated in the ENCODE database. We have also shown that the identified substitutions in P2 may affect the binding profile for this transcription factor. Chromatin looping was previously already associated to the regulation of *RUNX2* expression^34^. Also, DNase hypersensitivity mapping has recently allowed demonstrating a significant overlap between binding of the RUNX2 and/or the CTCF transcription factors on target genomic regions, as a highly dynamic regulatory mechanism driving early stages of osteogenic differentiation of mesenchymal progenitors^41^.

In addition, the P2 promoter overlaps with the locus of the *AL096865.1* lncRNA*,* showing an antisense orientation compared to that of the *RUNX2* gene. A recent paper identified *AL096865.*1 as a positionally conserved lncRNA located at a chromatin boundary within the locus, classifying it as a topological anchor point RNA (tapRNA)^33^. These tapRNAs have been shown to play a key role in the formation of regulatory chromatin loops and to exert a mutual positive regulation with the associated coding genes^33^. *AL096865.1* was found implicated in a complex chromosome rearrangement of chromosome 6 in a patient with cleidocranial dysplasia affecting *RUNX2* gene locus^42^. This is coherent with our results showing a positive correlation between the expression of *AL096865.1* and specific groups of *RUNX2* isoforms during the first days of osteogenic differentiation. Finally, chromatin looping was previously shown to regulate *RUNX2* expression during osteogenesis, also through interactions between the P1 promoter and the *SUPT3H* gene^34^.

Altogether, these data suggest that the evolutionary conserved sequence changes found within the P2/*AL096865.1* region of the locus, may have impacted *RUNX2* epigenetic regulation, through alterations in ZNF263 and/or CTCF binding and modulation of regulatory long distance DNA interactions within the genomic locus.

We originally demonstrated that also the expression of the *RUNX2-AS1* lncRNA, mapping on the reverse strand of the *RUNX2* ENST00000576263.5 coding isoform, is regulated during in vitro osteogenic differentiation of suture-derived cells. Other Authors have previously demonstrated that high levels of *RUNX2-AS1* in MSC isolated from patients with multiple myeloma correlated with a decreased osteogenic potential observed in these cells^43^. They showed that *RUNX2-AS1* acts as both a transcriptional and a post-transcriptional regulator of RUNX2, as it inhibits RUNX2 at both mRNA and protein expression levels, by interfering with its splicing^43^. Interestingly, *RUNX2-AS1* gene is located down-stream the proximal 3’UTR, and our analysis shows that the expression levels of *RUNX2-AS1* positively correlated with those of *RUNX2* isoforms with proximal 3’UTR.

Overall, these observations deserve further investigations to clarify the mechanisms of action of these two lncRNAs, also in light of the sequence changes that have affected their genomic loci during recent human evolution.

Our data also showed that the nucleotide substitutions fixed in the 3’UTR regions of the *RUNX2* gene locus of AMH may have led to changes in post-transcriptional regulation of *RUNX2* expression, by altering the binding of different miRNAs. To the best of our knowledge, there is currently a lack of information regarding the implication of post-transcriptional mechanisms of gene expression regulation in adaptive phenotypic evolution. We have indeed predicted that the *RUNX2* gene of AMH has either acquired or lost miRNA binding sites, compared to the corresponding loci of ancient hominins. We have specifically focused on the changes found in the *RUNX2* isoform containing the distal 3’UTR (namely, *ENST00000576263.5*). This variant is worth particular attention as it contains an extra terminal exon, which encodes an additional leucine-zipper DNA binding motif at the C-terminus of the protein. This domain makes this isoform potentially able to bind the target DNA with increased affinity. We speculate that this may enforce the transcriptional activation of bone-specific target genes involved in suture patterning and skull morphogenesis.

The miRNAs analysed in this study have not been previously annotated to be involved in the regulation of *RUNX2* expression^44,45^ or to play bone-related functions. Our results highlight miR-3150a-3p and miR-6785-5p as the best representatives for inferring functional implications. The expression trend of miR-3150a-3p inversely correlate with that of *RUNX2* total isoforms in CMSC during differentiation, in line with what have been observed in an another cellular model^46^. Also, the levels of miR-6785-5p inversely correlate with *RUNX2* transcripts during the in vitro osteogenic differentiation. Interestingly, the SPR-based analysis of the miRNA/target molecular interaction showed that miR-3150a-3p displays the higher affinity for the AMH’s *RUNX2* 3’UTR sequence, among the tested miRNAs. Conversely, this same miRNA failed to show any interaction with the target *RUNX2* sequence of ancient hominins. Conversely, miR-6785-5p showed a greater affinity for the ancient hominins sequence compared with the AMH’s *RUNX2* 3’UTR sequence. The modulation of *RUNX2* expression by these miRNAs could also involve indirect mechanisms, including additional modulators activated by the binding to the distal 3’UTR, which then affects the entire *RUNX2* transcript levels.

Moreover, our data suggest that the distal 3’UTR may be affected by additional different mechanisms of regulation. The expression trend of this isoform differs from the trend observed for the other groups of *RUNX2* isoforms, since its levels reached a peak at 10 days of osteogenic induction, whereas they decreased to basal levels afterwards.

To interpret these data in the light of the evolutionary changes observed in skull morphology, we may hypothesize that, besides the putative changes in epigenetic regulation proposed above, also the post-transcriptional regulation of *RUNX2* expression was modified over time. The sequence changes occurred in the 3’UTR have increased the affinity for inhibitory miRNAs in AMHs, leading to different timing of suture closure. This may have affected differently each skull suture, considering the chronological pattern of their fusion during skull morphogenesis. The metopic suture fuses first, in the perinatal period, being largely responsible for the shape of the frontal region. This site has a different developmental origin, as it is known to originate from the neural crest rather than from the mesoderm, as most of the skull, which seems to relate to different ossification programs in metopic and coronal sutures^47,48^. Indeed, craniosynostosis, the disease model considered in our study, is regarded as a paradigm developmental disorder due to defects in post-migratory neural crest cells^49^. The timing of metopic suture closure seems to strictly relate to the interconnected skull-brain evolution from nonhuman primates to modern humans. This is supposed to have integrated a complex network of feto-pelvic constraints and modification of frontal neurocranial ossification patterns, involving delayed fusion of the metopic suture^3^. The expression of Runx2 in neural crest-derived cells is indeed a crucial event driving the early stages of membranous bone ossification in the murine skull^50,51^. On this regard, metopic ossification has been recently shown to be more dependent on Runx2 expression compared to other areas of the skull of knockout murine models^52^. Moreover a recent work demonstrated that selected Runx2-targeting miRNAs may underlie the different osteogenic properties of osteoblasts and precursors isolated from frontal and parietal bones of murine skulls^53^. Therefore, we may speculate that the post-transcriptional modulation of *RUNX2* by miR-3150a-3p and miR-6785-5p has evolved in AMH as a site-specific mechanism enabling delayed metopic suture fusion during human skull patterning.

In conclusion, our data suggest that the genomic evolution of the *RUNX2* master bone gene, has introduced variations in regulatory sequences that may have affected its function, by both modifying chromatin structure and by acting at the transcriptional and post-transcriptional levels. According to our hypothesis, this may have affected skull shape during evolution, considering RUNX2 role in craniofacial development. Detailed in vivo studies in developing vertebrate models could provide a final validation of the hypotheses and predictions provided in this. Also, given that *RUNX2* promoter(s) drives its expression during osteogenesis of every bone in the body, additional hypothesis, including the effect of putative adaptive SNPs within skull-specific *RUNX2* enhancers, are also to be considered and worth investigating in future studies.

## Materials and methods

### RUNX2 locus alignment

*RUNX2* locus was aligned in AMH (Chr6:45,318,000–45,670,000, hg38) and ancient humans (Ancient Genome Browser, www.eva.mpg.de/neandertal/index.html and https://www.eva.mpg.de/genetics/genome-projects/denisova/index.html), using the Integrative Genomics Viewer (IGV) tool (2.3.72 version, software.broadinstitute.org/software/igv/), to map the sequence changes occurred during recent evolution^54,55^. We considered and annotated only nucleotide changes (single nucleotide variations, insertions and deletions) that differs from the reference sequence with a frequency greater than 79% of quality weighted reads. In addition, to assess if the nucleotide substitutions found within the promoters, the 3’UTR regions, and the two lncRNAs’ loci are specific to the evolution of AMH from ancient hominins, we also aligned the AMH genome with those of non-human Primates [chimpanzee (*Pan troglodytes*), gorilla (*Gorilla gorilla*), Sumatran orangutan (*Pongo abelii*), gibbon (*Nomascus leucogenys*) and rhesus macaque (*Macaca mulatta*)] using the UCSC Genome browser resources (https://genome.ucsc.edu).

### Surface plasmon resonance

The interaction between biotinylated DNA constructs (ligands) corresponding to the region of the distal 3’UTR containing the nucleotide changes fixed during evolution of AMH (5’-TGTCCCTCCCCAGCAGCGAAGCGCGCCCAGCGGAGG-3’, IDT Integrated DNA technologies, Coralville, IA, USA) or Neandertal/Denisovan (5’-TGTCCCTACCCAGCAGCGAAGCGCGCCCGGCGGAGG-3’, IDT Integrated DNA technologies) and DNA short sequences mimicking the different miRNA selected (analytes), were measured by SPR technique on a Biacore X100 instrument (Biacore, Uppsala, Sweden). Briefly, each biotinylated DNA sequence of AMH or Neandertal/Denisovan was immobilized on a Sensor Chip SA, pre-coated with streptavidin from Biacore AB. The capturing procedure on the biosensor surface was performed according to the manufacturer’s instructions and setting the aim for ligand immobilization to 1500 response units.

Running buffer was Hepes-buffered saline-EP, which contains 10 mM Hepes, pH 7.4, 0.15 M NaCl, 3 mM EDTA, 0.005% (v/v) Surfactant P20 (Biacore AB). Analytes were dissolved in running buffer, and binding experiments were performed at 25 °C with a flow rate of 30 µl/min. The association phase (k_on_) was followed for 180 s, whereas the dissociation phase (k_off_) was followed for 300 s. The complete dissociation of active complex formed was achieved by addition of 10 mM Hepes, 2 M NaCl, 3 mM EDTA, 0.005% (v/v) P20, pH 7.4, for 60 s before each new cycle start. Analytes were tested in a wide range of concentrations from 10 µM to 0.625 µM. When experimental data met quality criteria, kinetic parameters were estimated according to a 1:1 binding model, using Biacore X100 Evaluation Software and K_D_ value was determined using a global fit as the ratio k_off_/k_on_. Conversely, an affinity steady state model was applied to fit the data, when kinetic parameters were out of the range measured by the instrument but still a signal of interaction was detected. DNA short sequences mimicking miR-5589-5p and miR-4666a-5p have been used as positive and negative control respectively.

### Sample collection and cell isolation and culture

Calvarial tissue specimens were collected from unfused suture samples of patients undergoing cranial remodelling for nonsyndromic craniosynostosis, after obtaining their written informed consent (study protocol approved by the Ethical Committee of the Università Cattolica del Sacro Cuore, School of Medicine, number A/606/CE/2010 and 19056/14).

Calvarial-derived mesenchymal stromal cells (CMSC) were isolated in primary culture from suture tissue, as previously described^35,56^.

CMSC were cultured in standard growth medium composed as follow: Dulbecco's modified Eagle medium (DMEM) with high-glucose (Aurogene, Rome, Italy) supplemented with 1% l-glutamine (Euroclone, Milan, Italy), 1% antibiotics (penicillin 100 IU/ml, streptomycin 100 mg/ml; Euroclone), and 10% fetal bovine serum (FBS, Aurogene). Cells were grown at 37 °C in a humified atmosphere containing 5% CO_2_. Once isolated in primary culture, cells were sub-cultivated for multiple passages (P) and used at P2-P5 for subsequent analyses. CMSC were grown to confluence prior to the induction of osteogenesis. The osteogenic medium [DMEM low-glucose (Aurogene), 10% FBS (GIBCO by ThermoFisher Scientific, Waltham, MA, USA), 1% L-glutamine, 1% penicillin–streptomycin, dexamethasone 0.1 µM, β-glycerophosphate (Sigma Aldrich, Saint Louis, MO, USA) 10 µM and ascorbic acid 50 µM)], was changed every 3 days for until 21 days. CMSC cultured in standard growth medium were used as negative controls for differentiation (number of replicates = 5).

### RNA extraction and gene expression analysis

CMSC were lysed in TRIzol reagent (ThermoFisher Scientific) and then, total RNA was isolated using Direct-zol™ RNA Kit (Zymo Research, Orange, CA, USA) following the procedures described by the manufacturer, including a passage of DNA digestion with DNase I. Direct-zol™ kit assures highly efficient recovery of small RNAs including miRNAs. Isolated RNA was quantified using a UV spectrophotometer (DU 800 Beckman Coulter, Brea, CA, USA).

Up to 500 ng/µl of total RNA was reverse transcribed with GoScript™ Reverse Kit (Promega, San Luis Obispo, CA, USA) following the manufacturer’s protocol. Quantitative Real Time PCR (qPCR) using GoTaq(R) qPCR Master Mix (Promega) was performed to evaluate the expression of osteogenic markers at different time points, (*OSX* and *ALP*) and of the different *RUNX2* transcript variants by means of specific primer pairs^22^. Moreover, the expression of *AL096865.1* and *RUNX2-AS1* were assessed. Genes relative expression were obtained normalizing data to β-actin (ACTB) and results were calculated using 2^−ΔΔCt^ method^57^. The sequences of all primers used for each gene are provided in Supplementary File 5.

In parallel, miRNAs were reverse transcribed into cDNA using 15 ng/μl of total RNA and TaqMan™ Advanced miRNA cDNA Synthesis Kit (Applied Biosystems™ ThermoFisher Scientific). A synthetic miRNA (cel-miR-39-3p, mature miRNA sequence: UCACCGGGUGUAAAUCAGCUUG, ThermoFisher) was added to each sample of total RNA before retrotranscription, to a final concentration of 5 pM, to be assessed as an exogenous control for qPCR analysis. miRNA expression analysis was performed using TaqMan™ Fast Advanced Master Mix (Applied Biosystems™ ThermoFisher Scientific) under fast cycling conditions (StepOnePlus™ Real-Time PCR System, Applied Biosystems™). The levels of miR-3150a-3p, miR-6825-5p, miR-4700-5p, miR-149-3p, miR-4486 and miR-6785-5p were assessed using specific FAM-labelled TaqMan probes (Thermo Fisher Scientific) during in vitro osteogenic induction. Data were normalized to cel-miR-39-3p and expressed as relative quantity using 2^− ΔΔCt^ method.

### Statistical analysis

Data were analysed using GraphPad Prism software version 6.0 (San Diego, CA, USA). The histogram graphs were reported as means ± standard deviation (SD) of the mean. Unpaired Student’s t-test were performed for determining statistical differences between groups and probability values *(p)* < 0.05 were considered significant. Linear correlations were calculated using the Pearson’s correlation test considering a 95% confidence interval and setting the significant value at p = 0.05.

### Ethics approval

Human calvarial-derived stromal cells used in this study were isolated from surgical waste tissue specimens within the study protocol approved by the Ethical Committee of the Università Cattolica del Sacro Cuore, Faculty of Medicine and Surgery (protocol IDs A/606/CE/2010 and 19056/14), PI: Wanda Lattanzi.

All methods were carried out in accordance with relevant guidelines and regulations.

## Supplementary Information

Supplementary Information 1.

Supplementary Information 2.

Supplementary Information 3.

Supplementary Information 4.

Supplementary Information 5.

Supplementary File Descriptions.
