# Supplementary material for: Shaping modern human skull through epigenetic, transcriptional and post-transcriptional regulation of the RUNX2 master bone gene
Source: Sci Rep. 2021 Oct 29;11:21316. doi: 10.1038/s41598-021-00511-3 (PMC8556228; doi:10.1038/s41598-021-00511-3)
Supplement: Supplementary file 1 — Supplementary Information 1. [file 41598_2021_511_MOESM1_ESM.pdf]

chr6:45,318,000-45,670,000 (hg38)

RUNX2 Promoter 1	45327352/45328724	
RUNX2 Promoter 2	45419577/45422577	
AL096865.1	45421079/45422005	reverse strand
RUNX2-AS1	45573346/45576770	reverse strand
proximal 3'UTR	45547210/45551082	
distal 3'UTR	45663723/45664349	

POSITION	STRUCTURAL ELEMENT	NEANDERTAL	DENISOVA	SAPIENS	PRIMATES
45318008		C	C	T	
45318534			C	T	
45319630			C	T	
45320836		G	G	C	
45320850		G		A	
45325750		A	A	C	
45327733	*RUNX2 Promoter 1*	del T	del T		delT/insT
45329825			T	C	
45330745		A	A	G	
45332542		A		T	
45333210			G	A	
45334051		C	C	T	
45334180		C	C	T	
45334822			A	T	
45336829		T		C	
45338356		T	T	A	
45338634		G	G	A	
45339642		C	C	T	
45339706		T	T	C	
45340785		T		C	
45342075			C	T	
45342528		G	G	C	
45345105		G		A	
45346215		A		G	
45347758		ins T	ins T		
45348172			C	T	
45348931		A	A	C	
45349426		C	C	T	
45350356			G	C	
45350688		T	T	C	
45352139		T	T	C	
45352763		G	G	A	
45354631		del ACAC			
45356201		C		T	
45357302		T	T	C	
45357414		C	C	G	
45357663		A		T	
45357840		C	C	G	
45359664		T	T	G	
45360670		C	C	T	
45361311		A	A	C	
45361752		G	G	C	
45363325			A	G	
45365884		ins ATG	ins ATG		
45369713		G		C	
45369895		A		C	
45370199		T		G	
45370306		A		G	
45371563		A		C	
45372333		C	C	T	
45372336		C	C	T	
45373103			A	G	

45376267	C	C	T
45376961		C	T
45377511	C	C	A
45381240		G	C
45381662	C	C	T
45382370	G	G	A
45383654	G	G	A
45384961		A	C
45385130	A		G
45385262	T	T	C
45385363	T	T	C
45385693	A	A	T
45387872	C	C	T
45389962	C	C	A
45390006		del A	
45390676	G	G	A
45392139	C	C	A
45392696	C		T
45393473	T	T	C
45393561	T	T	C
45394137	T	T	C
45394318	C	C	T
45395819	C	C	G
45396059	A	A	C
45396380	C	C	T
45396533	G	G	A
45397776		G	T
45397950	C	C	T
45398191	A	A	G
45398269		T	C
45398470	C	C	T
45399209	G	G	A
45399880	C	C	T
45400061	G		A
45400719	G	G	T
45401060	A	A	G
45403160	C	C	A
45403816	A	A	T
45404316	ins A	ins A	
45404481	A	A	G
45405795	ins T	ins T	
45406106	A	A	T
45406446	G	G	A
45406681	A	A	G
45406871	A	A	T
45406962	T		A
45407920	T	T	A
45409122	C	C	T
45409301	A	A	G
45410040	G	G	A
45410111	T		C
45410355	G		A
45411236	G	G	A
45412387	C	C	A
45412910	G	G	T
45413345	G	G	A
45413662	T		C
45413847	A	A	G
45414033	A	A	T
45414187	T		C
45414356	T	T	C
45415341	C	C	A

45415684		ins C	ins C		
45415745			T	A	
45416281		C	C	T	
45416730		A	A	C	
45417013			C	T	
45417323		del T	del T		
45417702		T	T	C	
45417870		C		A	
45418005		A	A	G	
45418173		ins AAC	ins AAC		
45418359		T	T	C	
45419926	*RUNX2 Promoter 2*	A	A	G	G
45421406	*RUNX2 Promoter 2 - AL096865.1*	del T			delT
45421497	*RUNX2 Promoter 2 - AL096865.1*	G	G	A	G
45421552	*RUNX2 Promoter 2 - AL096865.1*	C	C	T	C
45422996		C	C	G	
45423101		ins CT			
45423212		C	C	G	
45423509			G	C	
45423553			A	C	
45423599		ins G			
45423917		C	C	G	
45424123		G	G	A	
45424206		G	G	T	
45424234		T	T	C	
45425245		G		A	
45425273		G	G	A	
45426515		T	T	C	
45428052		C	C	A	
45428055		C	C	A	
45428136		T	T	C	
45428183		G	G	A	
45428445		A	A	G	
45428807		C	C	T	
45431280		A	A	G	
45432409		G	G	A	
45436037		T	T	C	
45436792			T	C	
45437397		A	A	C	
45438792		G		T	
45440681		T		C	
45440703		G	G	T	
45441360			T	C	
45441620			insAGGA		
45444920		A	A	G	
45446805			C	A	
45449926		A	A	C	
45450665		T	T	C	
45451006		T	T	C	
45451691		C	C	A	
45451863			G	A	
45451909		A	A	G	
45452326		A		G	
45452430		A		G	
45453117		G	G	C	
45453311		C	C	G	
45453725		C		G	
45453893		T	T	C	
45456083		C	C	T	
45456182		G	G	A	
45456761		A	A	G	
45457203		G	G	A	

45459124	C	C	T
45459680	C	C	T
45459681	A	A	G
45460054	A	A	G
45460378	T	T	C
45460772	ins A		
45461402	A	A	C
45463151		C	T
45463635	A	A	T
45463931	T	T	C
45464477	A	A	G
45464528	T	T	C
45466314	del A		
45466408	T	T	G
45466475	T	T	C
45466670	T	T	G
45466728	C	C	T
45467721	G	G	A
45468512	T	T	C
45469767	A	A	G
45470735	G	G	A
45471286	T		C
45471949	G	G	A
45472241	A	A	G
45472874	A	A	T
45473906	T	T	G
45474646	T	T	G
45474980		A	G
45475343	T	T	A
45475439	G	G	A
45476521	G	G	A
45477230	T	T	A
45477247	T		G
45477791	A	A	G
45478314	T	T	G
45480606	T		C
45480607	G	G	A
45482473	C	C	T
45483648		T	C
45484099	T	T	C
45484452		A	G
45484561	C	C	T
45485646	T	T	C
45487458		G	T
45487529	T		C
45488434		T	C
45488716	ins A	ins A	
45489244	C	C	T
45489438		G	A
45489583	G	G	C
45492543	T	T	G
45493369		C	T
45493452		T	A
45493517	C		G
45493637		T	C
45493781	ins AC		
45494198	G	G	A
45494750	T	T	C
45496572	G	G	A
45497641	C		G
45498016	T	T	A
45499316	ins TC	ins TC	

45500028	G	G	A
45500047	C		T
45500319	A	A	G
45500337	G	G	T
45502379	C		T
45504561	C	C	T
45505000	T	T	C
45505352	C	C	T
45505361		del T	
45505519	C	C	T
45508018	G	G	T
45510288	A	A	G
45510379	A	A	C
45510719	A	A	T
45511532	T	T	A
45512762	C	C	G
45518433	C		T
45518470	G	G	C
45518646	A	A	T
45519849		T	A
45520207		A	C
45521021	C	C	T
45521193		T	A
45521964		del T	
45522445	A	A	C
45522555	G		A
45522975	T	T	C
45523302	G	G	A
45523624	A		T
45523631	del A	del A	
45524326	del T		
45524348	T	T	C
45525182		G	A
45525346	T	T	C
45527164		T	G
45527570		G	A
45528011	G	G	C
45528719	T	T	C
45529250		C	A
45529348		A	G
45529476	A		G
45530046		T	C
45531000	C	C	T
45531482	T		C
45531876	ins T	ins T	
45532263	A	A	G
45532733	A		G
45532876	A	A	G
45533427	del A	del A	
45533815	G	G	A
45534735	T		C
45534757		A	G
45535189		T	C
45535252		del A	
45539557	A	A	G
45539622	G		T
45540155		G	C
45541124	T		A
45542087		G	A
45542891		G	C
45543188		A	G
45543501		G	C

45543804		T	T	C	
45546169			C	T	
45546348		T		A	
45547352	*proximal 3'UTR*	T		C	C
45548181	*proximal 3'UTR*		A	T	T
45548438	*proximal 3'UTR*	T		A	A
45549013	*proximal 3'UTR*		C	T	T
45549121	*proximal 3'UTR*		G	C	C
45550352	*proximal 3'UTR*		C	A	C
45551578			T	C	
45551646		del T			
45552177		T		G	
45552293		T	T	A	
45552835		C	C	T	
45553554		T		G	
45553864			G	T	
45554255		G	G	C	
45554402		T		C	
45554459		T		C	
45555535			T	C	
45555675			G	A	
45556191		T	T	C	
45556210		ins A	ins A		
45557169			T	G	
45557209		G	G	A	
45557380		T		C	
45557850		T		C	
45557851			A	G	
45557902			C	A	
45558179			G	A	
45558472		C	C	T	
45558853			C	T	
45559395		A	A	G	
45560411		A		G	
45560484			C	T	
45561906		A		G	
45562240		A	A	C	
45562275		T		A	
45562302		C	C	T	
45562386			T	C	
45562484			C	T	
45562900			A	G	
45563656			del A		
45563827		A	A	T	
45564514		G	G	A	
45564862		G	G	C	
45565502		A	A	G	
45565524		C		T	
45566428		T	T	C	
45567382		A	A	G	
45567483		G	G	C	
45568258		T		C	
45568715		G	G	C	
45568873		G	G	C	
45569500		A	A	G	
45569598			A	G	
45570916		A		G	
45574592	*RUNX2-AS1*		C	A	A
45574880	*RUNX2-AS1*	T	T	A	T
45575744	*RUNX2-AS1*	A	A	G	G
45575775	*RUNX2-AS1*	C	C	A	C
45576233	*RUNX2-AS1*	G	G	C	G

45576631	*RUNX2-AS1*	G	G	A	A
45576849			G	A	
45578114		A	A	G	
45578238		C	C	T	
45578498		A	A	G	
45578920			G	A	
45579060		del T	del T		
45579297			G	A	
45579602			G	A	
45579653		ins T	ins T		
45580007			C	T	
45580627			T	C	
45581405		T		A	
45581411		ins TTC	ins TTC		
45581885			ins CTT		
45582220		T	T	C	
45582333		C	C	A	
45582845		C	C	T	
45583734		T	T	C	
45584548		T		A	
45585125		C	C	T	
45585524			G	C	
45585551		T		C	
45585721		del G	del G		
45585780		T	T	C	
45585905		C		G	
45587414		T	T	C	
45588332		A	A	G	
45588526		A	A	C	
45588554		A	A	G	
45589006			T	C	
45589763		G	G	T	
45590696		T	T	C	
45591129		T		C	
45591661			T	A	
45591741		G	G	A	
45592034		A		G	
45592248			A	G	
45592367		C		G	
45592995		C		T	
45593963		A	A	G	
45594062		T	T	C	
45594223		G	G	A	
45594559		A	A	G	
45594727		T	T	C	
45595889		C	C	G	
45595998		A	A	C	
45595999		C	C	G	
45596216			T	C	
45596540		T	T	G	
45596773		G	G	A	
45597217		A	A	G	
45597387		C	C	G	
45597418		G	G	A	
45597945		T	T	C	
45598205			T	C	
45598905			T	A	
45599690		T	T	G	
45600865		del G	delG		
45600984		C	C	A	
45602126		G	G	A	
45602312		T	T	C	

45602478	del T	delT	
45602633	ins C	insC	
45603530	C	C	T
45603568	A	A	G
45604334	A	A	G
45604762	A	A	C
45604842		G	A
45604992	ins T	insT	
45606156	A	A	G
45606612	A	A	T
45607102	G	G	A
45607392	G	G	C
45609050	del T	del T	
45609051	del A	del A	
45609061	C	C	T
45609107	C	C	T
45609397	C	C	T
45609465	G	G	T
45609670	C	C	T
45609777	A	A	C
45609912	C	C	A
45610870		A	G
45610941		delTT	
45612077		C	T
45612098	A	A	T
45612449	T	T	C
45613090	A	A	G
45614206	G	G	A
45615452	C	C	A
45616318	C	C	A
45616405	A	A	G
45617917	T	T	G
45618444	A	A	C
45619041	T	T	C
45619206	A	A	C
45619857	G	G	A
45620042	T	T	C
45620536		C	T
45621151	A	A	G
45621226	G	G	A
45621488	T	T	C
45621600	T	T	A
45621830	G	G	C
45621981	C	C	T
45622139		insT	
45622686	T	T	C
45622853	C	C	T
45623112	G	G	C
45623440	C	C	T
45623796		A	C
45624052	A		C
45624246	C	C	T
45624374	T	T	A
45624736	ins G		
45625352	C	C	A
45625682	G	G	A
45627148	C	C	T
45627396	del TTAG		
45627436	T	T	C
45627814	A	A	G
45627893		C	G
45628216		C	T

45629416		A	T
45629788	A	A	G
45630288	G	G	T
45630691	G	G	C
45630935	T	T	C
45630964	G	G	A
45630983	T	T	G
45631503	G	G	A
45631580	A	A	G
45631603	T		C
45631899	C	C	A
45632450		G	A
45632967	T	T	C
45633803	C	C	T
45634982	delA	C	A
45634984	C		T
45635908	A		G
45635909		A	G
45637485	A	A	C
45637608	A	A	C
45637619		A	G
45638162	G	G	C
45638248	T		C
45638518	G	G	A
45639465		T	C
45639528	T	T	C
45639882	G	G	A
45639887		T	A
45640090	G	G	T
45643430	G	G	A
45643945	A	A	G
45644351		C	T
45644435	A	A	C
45645577	C	C	T
45647917	A		G
45648044	insT	insT	
45648641	G	G	A
45648812	G	G	A
45649012	A	A	G
45649848	A	A	G
45649851	A		G
45650811	C	C	T
45650847	C	C	T
45650848		A	G
45651733	G	G	A
45652098	C	C	A
45652394	T	T	A
45652543	G		A
45653155	delC	delC	
45653405	G	G	A
45654214	G	G	C
45655011	A	A	T
45655358	T		C
45656258	A		T
45656321	C		G
45656851		T	C
45657364	G	G	A
45657659	A		G
45657806		T	G
45657988		T	G
45658383	T		C
45658385	C	C	T

45658535			G	A	
45658596		A	A	G	
45658776			A	C	
45658918			delT		
45659118		A		G	
45659159		C		T	
45659636		G	G	T	
45659713		A	A	C	
45660039		T		C	
45662956		A	A	T	
45663793	*distal 3'UTR*	A	A	C	C
45663814	*distal 3'UTR*	G	G	A	G
45665886		A	A	G	
45666355		C	C	A	
45666383		T		C	
45666763		G		T	
45666829		C	C	T	
45666846		A	A	T	
45667068		T	T	C	
45667173		C	C	G	
45667429		T		G	
45668242		T		C	
45668883		T		C	
