# Supplementary material for: Shaping modern human skull through epigenetic, transcriptional and post-transcriptional regulation of the RUNX2 master bone gene
Source: Sci Rep. 2021 Oct 29;11:21316. doi: 10.1038/s41598-021-00511-3 (PMC8556228; doi:10.1038/s41598-021-00511-3)
Supplement: Supplementary file 2 — Supplementary Information 2. [file 41598_2021_511_MOESM2_ESM.pdf]

motif_id	motif_alt_id	sequence_name	start	stop	strand	score	p-value	q-value	matched_sequence	
MA0528.1	ZNF263	neandertal	87	107	+	17.102	7.56e-07	0.00028	AGAGGTGGGGGTGGAGGTGGG	**Motif 2**
MA0528.1	ZNF263	denisova	88	108	+	17.102	7.56e-07	0.00028	AGAGGTGGGGGTGGAGGTGGG	
MA0528.1	ZNF263	sapiens	88	108	+	15.734	1.95e-06	0.000482	AGAGGTGGGGGTAGAGGTGGG	
MA0528.1	ZNF263	neandertal	147	167	-	6.367	0.000308	0.0109	AGGAGAGGAGGAAGGAAAATT	**Motif 3**
MA0528.1	ZNF263	denisova	148	168	-	6.367	0.000308	0.0109	AGGAGAGGAGGAAGGAAAATT	
MA0528.1	ZNF263	sapiens	148	168	-	4.959	0.000552	0.0186	AGGAGAGGAGGAAAGAAAATT	
MA0528.1	ZNF263	denisova	3	23	-	4.653	0.000624	0.0192	CAAAAAAAATGAGGAGGGGGG	**Motif 1**
MA0528.1	ZNF263	sapiens	3	23	-	4.653	0.000624	0.0192	CAAAAAAAATGAGGAGGGGGG	
MA0528.1	ZNF263	neandertal	2	22	-	4.204	0.000743	0.0199	CAAAAAAAATGAGGGGGGGGG	

FIMO (Find Individual Motif Occurrences): Version 5.4.1 compiled on Aug 25 2021 at 17:37:39

The format of this file is described at https://meme-suite.org/meme/doc/fimo-output-format.html.

fimo --oc . --verbosity 1 --thresh 0.001 MA0528.1.meme sequences.fa
