# Supplementary material for: Shaping modern human skull through epigenetic, transcriptional and post-transcriptional regulation of the RUNX2 master bone gene
Source: Sci Rep. 2021 Oct 29;11:21316. doi: 10.1038/s41598-021-00511-3 (PMC8556228; doi:10.1038/s41598-021-00511-3)
Supplement: Supplementary file 3 — Supplementary Information 3. [file 41598_2021_511_MOESM3_ESM.pdf]

Supplementary File 3 Selected miRNAs for binding to proximal and to distal 3'UTRs

**microRNA**	**Mature sequence***
**Proximal 3'UTR**	
**miR-3118**	U**GUGACUG**CAUUAUGAAAAUUCU
**miR-134-5p**	U**GUGACUG**GUUGACCAGAGGGG
**miR-5002-3p**	U**GACUGCC**UCACUGACCACUU
**miR-5006-5p**	U**UGCCAGG**GCAGGAGGUGGAA
**miR-4666a-5p**	A**UACAUGU**CAGAUUGUAUGCC
**miR-299-3p**	U**AUGUGGG**AUGGUAAACCGCUU
**miR-3143**	**AUAACAUU**GUAAAGCGCUUCUUUCG
**miR-6840-3p**	G**CCCAGGA**CUUUGUGCGGGGUG
**miR-6809-5p**	U**GGCAAGG**AAAGAAGAGGAUCA
**Distal 3'UTR**	
**miR-8089**	C**CUGGGGA**CAGGGGAUUGGGGCAG
**miR-4667-5p**	A**CUGGGGA**GCAGAAGGAGAACC
**miR-4700-5p**	U**CUGGGGA**UGAGGACAGUGUG
**miR-6825-5p**	U**GGGGAGG**UGUGGAGUCAGCAU
**miR-3150a-3p**	C**UGGGGAG**AUCCUCGAGGUUGG
**miR-6763-5p**	C**UGGGGAG**UGGCUGGGGAG
**miR-3126-5p**	U**GAGGGAC**AGAUGCCAGAAGCA
**miR-6875-5p**	U**GAGGGAC**CCAGGACAGGAGA
**miR-6883-5p**	A**GGGAGGG**UGUGGUAUGGAUGU
**miR-6785-5p**	U**GGGAGGG**CGUGGAUGAUGGUG
**miR-149-3p**	A**GGGAGGG**ACGGGGGCUGUGC
**miR-4728-5p**	U**GGGAGGG**GAGAGGCAGCAAGCA
**miR-4486**	G**CUGGGCG**AGGCUGGCA

***The underlined sequence represents the seed region.**
