# Supplementary material for: Shaping modern human skull through epigenetic, transcriptional and post-transcriptional regulation of the RUNX2 master bone gene
Source: Sci Rep. 2021 Oct 29;11:21316. doi: 10.1038/s41598-021-00511-3 (PMC8556228; doi:10.1038/s41598-021-00511-3)
Supplement: Supplementary file 4 — Supplementary Information 4. [file 41598_2021_511_MOESM4_ESM.pdf]

Alignment of microRNAs sequences of AMH (Sapiens), Neandertal and Denisova species

- **miR-3143**

SAPIENS
TAG**<u>ATAACATT</u>**GTAAAGCGCTTCTTTCGCGGTTGGGCTGGAGCAACTCTTTACAATGTTTCTA

DENISOVA
TAG**<u>ATAACATT</u>**G**C**AAAGCGCTTCTTTCGCGGTTGGGCTGGAGCAACTCTTTACAATGTTTCTA

T->C 100%

Coordinates (GRCh38)
chr6: 27147626-27147688 [+]

- **miR-149-3p**

SAPIENS
GCCGGCGCCCGAGCTCTGGCTCCGTGTCTTCACTCCCGTGCTTGTCCGAGGAGGGA**<u>GGGAGGG</u>**ACGGGGGCTG
TGCTGGGGCAGCTGGA

NEANDERTHAL
GCCGGCGCCCGAGCTCTGGCTCCGTGTCTTCACTCCCGTGCTTGTCCGAGGAGGGA**<u>GGGAGGG</u>**ACGGGGGCTG
TGCTGGGGC**G**GC**C**GGA

A->G 97%
T->C 94%

DENISOVA
GCCGGCGCCCGAGCTCTGGCTCCGTGTCTTCACTCCCGTGCTTGTCCGAGGAGGGA**<u>GGGAGGG</u>**ACGGGGGCTG
TGCTGGGGCAGC**C**GGA

T->C 100%

Coordinates (GRCh38)
chr2: 240456001-240456089 [+]
