# Supplementary material for: Shaping modern human skull through epigenetic, transcriptional and post-transcriptional regulation of the RUNX2 master bone gene
Source: Sci Rep. 2021 Oct 29;11:21316. doi: 10.1038/s41598-021-00511-3 (PMC8556228; doi:10.1038/s41598-021-00511-3)
Supplement: Supplementary file 5 — Supplementary Information 5. [file 41598_2021_511_MOESM5_ESM.pdf]

Gene	Primer sequence
β-actin (ACTB)	Forward: TCGTGCGTGACATTAAGGAG
	Reverse: CCATCTCTTGCTCGAAGTCC
Sp7 transcription factor (osterix, OSX)	Forward: ATCCAGCCCCCTTTACAAGC
	Reverse: TAGCATAGCCTGAGGTGGGT
Alkaline phosphatase (ALP)	Forward: CCGTGGCAACTCTATCTTTGG
	Reverse: GCCATACAGGATGGCAGTGA
RUNX2 total isoforms	Forward: GAACCCAGAAGGCACAGACA
	Reverse: GGATGAGGAATGCGCCCTAA
RUNX2 P1-derived isoforms	Forward: AGCACAGTGACACCATGTCA
	Reverse: GGGCTCACGTCGCTCATTT
RUNX2 P2-derived isoforms	Forward: ATGCGTATTCCCGTAGATCC
	Reverse: GGGCTCACGTCGCTCATTT
RUNX2 isoforms with first 3'UTR	Forward: AGACATGCTGCAGGTCCTC
	Reverse: AAAATAATCTATCCTGATCAGTTA
RUNX2 isoforms with second 3'UTR	Forward: AGAGGAGCTCAAAGCAAGTC
	Reverse: TGTGAACGTCACCAGAGATG
AL096865.1	Forward: TGCAAAGCTCCCCTGTCTC
	Reverse: CAGCGGCTGCGATTCGCA
RUNX2-AS1	Forward: TACTTCCCAGACCAGACCACA
	Reverse: CCTGGCTATTGCTTTTGAGGC
