# Supplementary material for: Shaping modern human skull through epigenetic, transcriptional and post-transcriptional regulation of the RUNX2 master bone gene
Source: Sci Rep. 2021 Oct 29;11:21316. doi: 10.1038/s41598-021-00511-3 (PMC8556228; doi:10.1038/s41598-021-00511-3)
Supplement: Supplementary file 6 — Supplementary File Descriptions. [file 41598_2021_511_MOESM6_ESM.docx]

**Supplementary File 1** The table reports all the changes annotated in the *RUNX2* locus (Chr6:45,318,000-45,670,000 hg38) by aligning AMH and ancient species’ (Neandertal and Denisovan) genomes. Selected changes map within the P1 and P2 promoter sequences, and in both proximal and distal 3’UTRs. The nucleotide changes occured in *AL096865.1* and *RUNX2-AS1* gene loci are also highlighted. The table also displays the nucleotide changes annotated in Primate species compared with Homo sapiens, mapping in *RUNX2* promoters and 3’UTRs, and in both *AL096865.1* and *RUNX2-AS1*.

**Supplementary File 2** Analysis of the position-specific scoring matrices for ZNF263 (Id MA0528.1) at *RUNX2* P2 region for the three species (Neandertal, Denisova and Sapiens) using FIMO. The table shows the matrix score at the most significative occurrences and the sequence variants at the identified motifs (Motif 1, 2 and 3). The FIMO software version and parameters used are indicated below the table.

**Supplementary File 3** The table shows all the miRNAs with their mature sequence whose seed sequences span the regions that include one of the substitutions fixed in the proximal and distal 3’UTRs of *RUNX2* of Homo sapiens compared to ancient hominines. The seed region sequence is underlined.

**Supplementary File 4** The file reports the nucleotide changes (in bold text) with the respective frequencies, occurred in the AMH miR-3143, compared with the Denisovan sequence, and in the AMH miR-149-3p, compared both with Neandertal and Denisovan species. The identified substitutions map outside the seed sequences, marked in bold and underlined for each miRNA.

**Supplementary File 5** The table reports the sequences of all the oligonucleotide primer pairs (forward and reverse) used for each gene analysed (see text for additional details).
